# Evaluation of an intelligent wheelchair system for older adults with cognitive impairments

**DOI:** 10.1186/1743-0003-10-90

**Published:** 2013-08-07

**Authors:** Tuck-Voon How, Rosalie H Wang, Alex Mihailidis

**Affiliations:** 1 The Institute of Biomaterials and Biomedical Engineering, University of Toronto, Toronto ON, Canada; 2 Department of Occupational Science and Occupational Therapy, University of Toronto, Toronto ON, Canada; 3 Toronto Rehabilitation Institute, University Health Network, Toronto ON, Canada; 4 Intelligent Assistive Technology & Systems Lab, University of Toronto, Toronto ON, Canada

**Keywords:** Aging, Assistive technology, Cognitive impairments, Computer vision, Dementia, Intelligent wheelchair, Mobility, Older adult, Powered wheelchairs, Smart wheelchair

## Abstract

**Background:**

Older adults are the most prevalent wheelchair users in Canada. Yet, cognitive impairments may prevent an older adult from being allowed to use a powered wheelchair due to safety and usability concerns. To address this issue, an add-on Intelligent Wheelchair System (IWS) was developed to help older adults with cognitive impairments drive a powered wheelchair safely and effectively. When attached to a powered wheelchair, the IWS adds a vision-based anti-collision feature that prevents the wheelchair from hitting obstacles and a navigation assistance feature that plays audio prompts to help users manoeuvre around obstacles.

**Methods:**

A two stage evaluation was conducted to test the efficacy of the IWS. *Stage One: Environment of Use* – the IWS’s anti-collision and navigation features were evaluated against objects found in a long-term care facility. Six different collision scenarios (wall, walker, cane, no object, moving and stationary person) and three different navigation scenarios (object on left, object on right, and no object) were performed. Signal detection theory was used to categorize the response of the system in each scenario. *Stage Two: User Trials* – single-subject research design was used to evaluate the impact of the IWS on older adults with cognitive impairment. Participants were asked to drive a powered wheelchair through a structured obstacle course in two phases: 1) with the IWS and 2) without the IWS. Measurements of safety and usability were taken and compared between the two phases. Visual analysis and phase averages were used to analyze the single-subject data.

**Results:**

*Stage One:* The IWS performed correctly for all environmental anti-collision and navigation scenarios. *Stage Two:* Two participants completed the trials. The IWS was able to limit the number of collisions that occurred with a powered wheelchair and lower the perceived workload for driving a powered wheelchair. However, the objective performance (time to complete course) of users navigating their environment did not improve with the IWS.

**Conclusions:**

This study shows the efficacy of the IWS in performing with a potential environment of use, and benefiting members of its desired user population to increase safety and lower perceived demands of powered wheelchair driving.

## Background

### Older adults and powered wheelchairs

As older adults age, there is a greater likelihood that they will develop chronic conditions that negatively impact their mobility, including arthritis, osteoporosis, or heart disease. Over time these chronic conditions can worsen and lead to difficulty in walking or to adverse events that may prevent walking altogether (for example, a fall or stroke). Older adults who are unable to walk are at risk of losing their independent mobility, an important aspect of their quality of life [1]. Fortunately, wheelchairs (both manual and powered) and scooters exist to compensate for this loss of walking ability and to offer a needed means of independence. It is known that older adults are the most prevalent users of wheelchairs in Canada, and it is estimated that 49% of older adults in Canadian institutional settings use a wheelchair [2]. With the demographic shift to a larger proportion of older adults, the demand for wheelchairs is expected to grow as well.

Powered wheelchairs in particular, are used by older adults who do not have the physical ability to propel a manual wheelchair or use a scooter. The benefits of powered wheelchair use have been documented: allowing older adults the freedom to engage with their environment and also relieving burden on caregivers [3]. However, a certain skill set is required to be able to operate a powered wheelchair, including: the ability to adequately control the driving interface; good reasoning to interpret and act upon the driving situation; sensory awareness of the environment; and adequate memory, attention, and focus [4]. Yet physical, sensory, and cognitive impairments could hamper an older adult’s ability to meet such requirements.

The impact of cognitive impairments on powered wheelchair use may be most prevalent within institutional settings. In Canada, it is estimated that approximately 65% of older adults in these settings have cognitive impairments [5]. Cognitive impairments could lead to agitation, poor impulse control, poor executive reasoning and planning, impaired attention, and memory difficulties [6]. The result of these deficits is an increased risk for accidents and difficulty in performing driving tasks. There is concern that older adults with cognitive impairments could cause collisions or accidents that harm themselves, others, or property. In many institutions, this concern has barred the use of powered wheelchairs entirely, whereas other institutions have applied screening procedures to judge safe and proper driving [7]. However, if an older adult cannot demonstrate competency with a powered wheelchair, then they are prevented from having a means of independent mobility.

### Intelligent/smart wheelchair research

Intelligent or smart wheelchairs have been in development to increase the accessibility and safety of powered wheelchairs for individuals with physical, sensory, or cognitive impairments. Over the past three decades researchers have investigated different input devices, environmental sensors, and control schemes to augment powered wheelchairs [8]. Although novel technology has been developed, much of this technology has not been transferred to the consumer market. One reason for this is the lack of user involvement in the design and testing of many intelligent wheelchairs [9]. Evidence to show the usefulness of the technology with its desired user population is a necessary component of technology transfer.

Since Simpson’s review of intelligent wheelchairs [8], more projects have included user testing in their evaluation or iterative design. However, only a subset of intelligent wheelchairs have been tested with individuals who have cognitive impairments (see Table 1, [9-17]), and fewer still, with cognitively impaired older adults.

**Table 1:** Previous intelligent wheelchairs evaluated with cognitively impaired individuals

**Wheelchair**	**Sensors**	**Control scheme**	**Wheelchair description**	**User interface**	**Clinical populated tested**	**Study design**	**Study outcomes**
Hephaestus[11,12]	Sonar and bumper	Semi-autonomous	Wheelchair attempts to automatically steer around obstacles, or will stop before hitting an obstacle.	Joystick	Able bodied and disabled individuals. 3 had cerebral palsy, 1 with post-polio syndrome. Unknown ages.	Participants drove through three short obstacle tasks with the wheelchair’s navigation assistance and without assistance (4 times for each scenario). Objective driving performance (time/ collisions) and subjective preference was recorded.	Wheelchair’s navigation assistance was preferred by disabled individuals over no help. Navigation assistance increased the time needed to drive through the courses and collisions still occurred with navigation assistance. Findings were limited due to the short evaluation period (1 day) and lack of complexity in the obstacle courses.
Smart Wheelchair (UK Call Centre) [9,10]	Infrared line follower and bumper	Semi-autonomous	Wheelchair has ability to follow a line on the floor, bumpers provide anti-collision function.	Various input controls supported (e.g., switch, joystick)	Children with physical/cognitive impairments. A number of studies, including a test with 4 children who have cerebral palsy. Age 5–13.	Participants received training with the Smart Wheelchair for two 1-hour sessions per week, 8 weeks. Children progressed from single-room to school environments. Driving skills and psychosocial outcomes were measured.	3 of 4 children were able to develop 3 or more independent driving skills, and parents also reported positive changes in child’s confidence, motivation and affect. Trainers were able to decrease the assistance of wheelchair as the child showed progress in driving skills.
PALMA [13]	Sonar	Fully autonomous or semi-autonomous	Sonar sensors prevent the vehicle from hitting an object. Fully autonomous mode: PALMA navigates with no user input and has no set course. Semi-autonomous: the user has various levels of control over starting/stopping and direction of travel.	4 directions and 1 stop button. Visual (LED) and audio feedback for collisions.	Children with neuromotor disorders. Tested with 5 children with various levels of cognitive impairments. Age 3–7.	Children completed multiple 15-min driving sessions, which included driving around a room and goal oriented tasks (i.e. driving through door frames). Degree of help given by the wheelchair was lowered as a child showed proficiency. An average of 6 sessions per child.	PALMA was considered a successful training/rehabilitation tool. Its various levels of autonomy allow personalized customization to a child’s impairments. All children improved to need less assistance after several sessions.
CWA (Collaborative Wheelchair Assistant) [14]	Barcode scanner and wheel odometers (for positioning)	Semi-autonomous	Wheelchair travels along preset paths (barcodes are used to define paths in environment). User can use the joystick to avoid unexpected obstacles along those paths, and then be automatically steered back to the preset path.	Joystick	Individuals with motor/cognitive impairments. Tested with 3 individuals with cerebral palsy, and 2 individuals with traumatic brain injury individuals. Age 16–48.	Participants were trained on 6 driving tasks and then navigated through a short path with fixed obstacles. 10 path sessions for each participant, alternating between wheelchair assistance and no assistance. Collisions and joystick motion were recorded.	CWA assistance was able to help users navigate through the course with no collisions. The large variability in patient impairments showed a need for adaptable interfaces. When assistance was enabled, less joystick motion was needed and it was inferred that this relaxed the driving task.
Intelligent Wheelchair (University of Zaragoza) [15]	Planar laser and wheel odometers	Semi-autonomous	Wheelchair dynamically detects obstacles in the environment and offers to the user directions of travel that will avoid the obstacles.	Custom touch and visual interface.	Young adults with cognitive impairments. Tested with 4 students with cerebral palsy. Age 11–16.	Participants were trained to use the interface first through a computer simulation (45-60 min). Field trials consisted of driving in an uncontrolled school environment (1 session, 1 week after training). Metrics on task performance and user behavior were recorded.	Overall users were able to drive through the school environment. 6 collisions occurred that needed external intervention. Reasons for collision included obstacles at lower height of laser and system errors. The degree of cognitive impairment increased the time of driving and decreased the proficiency with the interface.
Anti-collision Skirt [16]	Low force contact sensor skirt	Semi-autonomous	Contact sensor skirt will stop the wheelchair from moving towards an object when pressure on the skirt is detected.	Joystick	Older adults with cognitive impairments. Tested with 6 older adults with mild dementia. Age 65 + .	Multiple single-subject studies, where each participant was evaluated at baseline (manual wheelchair), training (12 1-hour training sessions), and extended power wheelchair use (if deemed suitable after training). Measures of safety and mobility were taken from perception of users and external caregivers in a nursing home.	Wheelchair stopped before serious collisions occurred. False or missed collisions occurred due to gaps in the skirt, bumps on the floor, or objects above the skirt. Reception and use of the wheelchair was mixed. One adult improved mobility and well-being, another did not like its usability, slow speed, and bulky appearance. Other residents were not suitable for extended use.
CARMEN (Collaborative Autonomous Robot for Mobility Enhancement) [17]	Planar laser and wheel odometers	Semi-autonomous	Wheelchair and user share control of direction at the same time. Direction output is based on sensor readings and user input.	Joystick	Various evaluations with adults. Recently tested: 18 (mostly older) adults with physical/cognitive disabilities. Age 36–84.	Participants drove the wheelchair through a household course under two conditions: 1) standalone mode – which prevents collisions, and 2) collaborative mode – where the user and wheelchair share control. At least one run in each condition.	Not all users could complete the course in standalone mode, but all users completed it in collaborative mode. Generally collaborative mode was more efficient, unless users had high cognitive ability, in which case they may have fought the assistance that the wheelchair was giving.

This table summarizes previous work on intelligent wheelchairs for individuals with cognitive impairment. Comparisons can be made to our design in terms of technology used and the way the wheelchair was evaluated.

### Research purpose

The overarching goal of this project was to develop and have users evaluate an intelligent system that is meant to help older adults with cognitive impairments drive a powered wheelchair safely and effectively. It is proposed that this project will give greater insight into the design of intelligent wheelchairs for this population.

The purpose of the study described in this paper is to evaluate an intelligent wheelchair in terms of its efficacy to perform within a potential environment of use, and efficacy to provide safety and usability for its users.

### Overview of the Intelligent Wheelchair System (IWS)

The Intelligent Wheelchair System (IWS) is design by the Intelligent Assistive Technology & Systems Lab (IATSL, University of Toronto) as an add-on to existing powered wheelchairs [18,19]. Its purpose is to promote the safety and usability of powered wheelchairs for older adults with cognitive impairments. To achieve this purpose, the IWS adds two features when attached to a powered wheelchair: 1) anti-collision – to gently stop the wheelchair before it hits obstacles; and 2) navigation assistance – to help the user manoeuvre around obstacles in their environment by playing audio prompts that suggest navigation directions. For driving, a semi-autonomous control scheme is used, where the user retains control of the wheelchair in most situations, but is prevented from entering into collision situations and is aided (prompted) if they cannot navigate to avoid obstacles after a certain amount of time. This scheme was chosen because the authors believe that it would promote user enablement, exploration, and learning. The technology is meant to support the driver’s capabilities, not to replace the driver, a viewpoint that has been argued for by Nisbet [9].

The IWS is a continuation of intelligent wheelchairs designed by IATSL. Our previous systems have used a variety of sensors for obstacle detection, including: a mechanical contact skirt [16], an infrared sensor [20], and a stereovision camera [21]. Results from our previous work and a review of the field [8] suggest that vision is a promising sensor modality for detecting obstacles due to its large field of view (especially in terms of height, which is a limitation of planar lasers and sonar), low price point, and robustness over infrared to lighting noise. The IWS builds upon our previous work by improving the anti-collision algorithm and performance of the system, the details of which are presented over the next three sections. A distinguishing mark of our system, compared to other intelligent wheelchairs that have been tested with cognitively impaired individuals, is the depth (3D) vision sensor and the audio direction feedback (see Table 1 for technology comparison).

### IWS: hardware implementation

**Figure 1:**
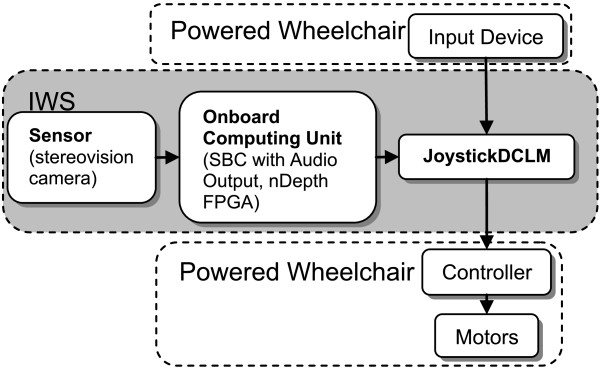
Hardware diagram of the Intelligent Wheelchair System (IWS) when attached to a powered wheelchair.

Figure 1 shows a block diagram of the IWS. A sensor is used to detect the environment in front of the wheelchair. This environmental information is then sent to an Onboard Computing Unit (*OCU*) for processing and analysis. The *OCU* determines the distance of obstacles from the front of the powered wheelchair and the free space surrounding those obstacles. If an obstacle is too close to the powered wheelchair the *OCU* will issue a prevention command to the *joystickDCLM* (Direction Control Logic Module). From this, the *joystickDCLM* will prevent any further wheelchair movement towards the obstacle. The *joystickDCLM* is situated between the user’s input device (i.e. joystick) and the wheelchair’s controller; it has the ability to prevent unsafe user inputs from reaching the wheelchair’s motors. When the wheelchair remains stopped by an obstacle for a certain period of time (~2 seconds), the *OCU* will play an audio prompt to help the user navigate into free space surrounding the obstacle (e.g., “try turning left”, “try turning right”). This audio prompt is repeated every five seconds if the wheelchair continues to remain stopped.

A FocusRobotic’s nDepth™ stereovision camera is used as the sensor. This camera was chosen because of its adequate field of view (64° horizontal FOV, 41° vertical FOV) and ability to produce depth/distance data at real-time speeds (30 Hz, 720x480 resolution) in its corresponding nDepth™ FPGA (field-programmable gate array) board. The FPGA board is housed within the *OCU*, and outputs depth data to a 1.8 MHz Pentium M single board computer (SBC). This SBC performs further image analysis (see “IWS: software implementation” section below), and is connected to speakers for audio output, as well as the *joystickDCLM* via an RS-232 link. The *joystickDCLM* is comprised of a microcontroller (Atmel ATmega644p) and has two A/D-D/A (analogue/digital-digital/analogue) channels for interfacing with the input device and the wheelchair’s controller. Communication between the *OCU* and the *joystickDCLM* utilizes a custom serial protocol through the RS-232 link.

### IWS: software implementation

**Figure 2:**
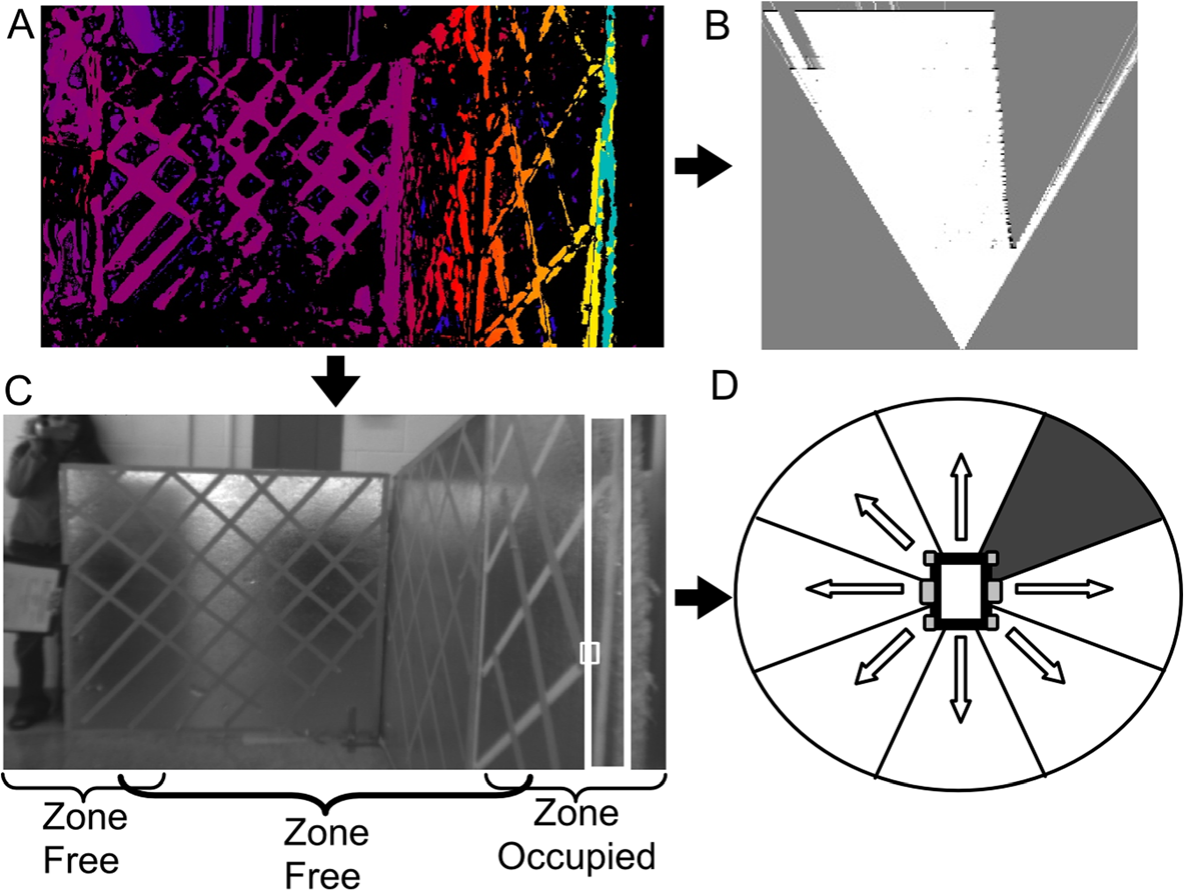
**Summary of software operations performed within the IWS. A)** Depth image is produced by the nDepth™ FPGA; brighter regions are closer to the sensor. **B)** Top-down occupancy grid (created from the depth image) is used for navigation prompting; white is free space, grey is unknown, small black regions in between the grey and white space are occupied. **C)** Regions of high disparity are identified and bounded with white rectangles, the zone they occupy is noted. In this case an obstacle occupies the forward-right zone. **D)** Zones with obstacles occupying them are blocked by the joystickDCLM (Direction Control Logic Module). In this case the forward-right movement is prevented.

When the SBC receives a depth image from the FPGA (Figure 2A) it performs two operations. One operation is to convert the depth image into a top-down occupancy grid of the environment (Figure 2B). The algorithm for this takes the maximum disparity (i.e. closest depth) of each column in the depth image and performs ray tracing to map these points into their corresponding world location. For a detailed description of this algorithm, the reader is referred to an earlier paper [20]. The other operation is to analyze the depth image for conjoined pixels (blobs) of high disparity, and create bounding boxes around blobs greater than a minimum size threshold (Figure 2C); these blobs are assumed to be obstacles in the environment. Disparities that are bounded relate to the desired stopping distance from obstacles. For example, if the stopping distance is 700 mm, the disparities of interest include values related to 700 mm and closer. A minimum size threshold was used in order to eliminate the effects of noise (both local and random) within the depth image, while still maintaining the ability to detect small objects (for example, canes). This method of noise reduction has been described by Murray and Little [22].

When a blob is bounded, the SBC determines which zone it occupies in the image. With the current sensor’s FOV, the image is segmented into three zones that each corresponds to a different powered wheelchair motion (i.e., forward-left, forward, and forward-right). Once a zone is occupied, the SBC sends a serial command to the *joystickDCLM* to prevent the corresponding powered wheelchair motion (Figure 2D). When the zone is free, this command is reversed. If the wheelchair remains stopped by an obstacle for a set duration, the left and right sides of the occupancy grid (Figure 2B) are analyzed to determine which side has the greatest free space, and the outcome of this calculation determines the audio prompt’s direction played to the user.

### IWS: mounting and latest improvements

**Figure 3:**
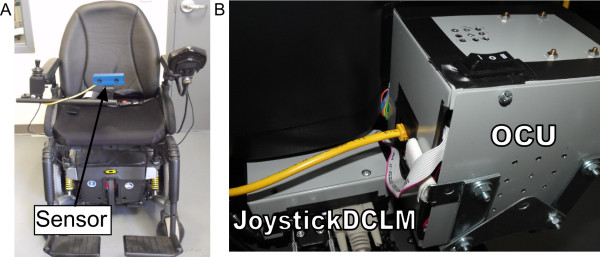
**IWS mounting on a powered wheelchair. A)** The sensor is mounted on a swing-able arm on the front of the powered wheelchair. **B)** The joystickDCLM (Direction Control Logic Module) and OCU (Onboard Computer Unit) are mounted behind the wheelchair seat.

Figure 3, shows the IWS as it is mounted onto a powered wheelchair. Compared to our last iteration [21], the latest IWS has a new vision algorithm for detecting obstacles in the environment and the capability of preventing specific directions of wheelchair movement. The latest IWS also has significantly improved sensor coverage (50° to 64° horizontal FOV), image resolution (320x240 to 720x480), system update rate (2.25 to 24.47 Hz), and occupancy grid detail resolution (10 cm to 1 cm). As well, the system has been miniaturized to mount on the back of a powered wheelchair.

## Methods

The objective of this research was to evaluate the efficacy of the IWS in terms of real-world use for older adults with cognitive impairments. There were two stages of evaluation: 1) efficacy within a potential environment of use, and 2) efficacy with members of the desired user population.

### Stage one: environment of use

The goal of stage one was to evaluate the IWS’s anti-collision and navigation assistance features within a potential environment of use: institutional homes for the elderly. Trials within an experimental environment (with fixed fluorescent lighting) were designed to test the IWS’s ability to perform with common real-world objects from this setting. If the IWS proved capable of performing well with these objects, then it has the potential to be deployed in the same real-world setting. Similar trials were conducted with a previous iteration of the system [21] and results from those trials were used as a basis of comparison to evaluate the impact of the improvements made to the IWS.

**Figure 4:**
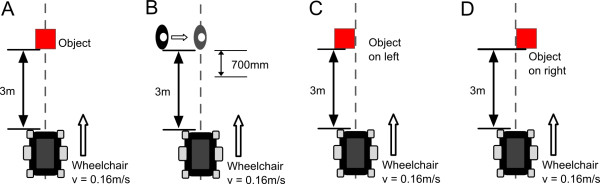
**Environment of use testing scenarios.** Anti-collision and navigation are tested by driving the wheelchair towards different objects. A 3 m distance was set in order for the wheelchair to achieve a constant velocity of 0.16 m/s. For anti-collision testing: **A)** stationary objects on centerline; **B)** moving person that steps onto the centerline when wheelchair is within 700 mm of the person. For navigation testing: **C)** object on left of centerline; **D)** object on right of centerline.

To evaluate the IWS’s anti-collision feature, the IWS was mounted on a Pride Mobility™ Quantum 6000z powered wheelchair and driven towards six different object scenarios that would typically be found in a long-term care facility: 1) white wall pillar, 2) aluminum four-wheeled walker, 3) aluminum walking cane, 4) stationary person, 5) moving person, and 6) no object (to test for false detections). The wheelchair was driven from a distance of 3 m toward the objects to allow for a constant velocity of 0.16 m/s to be reached (Figure 4A). This velocity was chosen to allow for comparison with previous testing results [21]. Driving motion continued until the anti-collision feature stopped the wheelchair or the wheelchair hit the object. A threshold of 700 mm was set as the anti-collision stopping distance from the front of the sensor; this value was chosen to compare with previous testing results, and was inferred as a safe stopping distance with the camera mounted 300 mm behind the furthest forward point of the wheelchair (i.e. the footrests). For the moving person scenario, the person remained outside the field of view (FOV) of the camera until the camera came within 700 mm of the person (Figure 4B). At this time, the person would step into the FOV and stop in front of the wheelchair; this represented the worst case scenario of an object entering the FOV of the camera for the set threshold distance. The wheelchair was driven toward each object scenario 20 times. Both the response of the anti-collision feature and the stopping distance from the front of the sensor to the object were recorded.

To test the IWS’s navigation feature, the same wheelchair was driven toward three different navigation scenarios: 1) an object placed left of centre (Figure 4C); 2) an object placed right of centre (Figure 4D); and 3) no object (to detect false prompting). Each scenario was repeated 20 times and the object used in testing was the four-wheeled walker. Driving motion was allowed to continue until the wheelchair stopped and prompted the user, or the wheelchair hit the object. In each scenario, the audio prompt given by the navigation feature was recorded.

Signal detection theory was used to group the responses from the anti-collision and navigation assistance features. The categories of responses were: 1) Hit (object present, object detected/correct prompt issued); 2) Miss (object present, no object detected/absent or incorrect prompt); 3) False Alarm (no object present, object detected/prompt issued); and 4) Correct Reject (no object present, no object detected/no prompt issued).

### Stage two: user trials

#### ***Overview & subjects***

The goal of stage two was to test if the IWS could improve the safety and usability of a powered wheelchair when driven by older adults with cognitive impairments. For this, a comparative approach was taken to evaluate the safety and usability of driving a powered wheelchair with and without the IWS.

Due to the difficulty in recruiting large numbers of the clinical population and the desire not to overlook the effects of the IWS on the individual, a single-subject research design was chosen. In single-subject research, each participant acts as their own control. For a single-subject research design, repeated and frequent outcome measures of a participant are taken in a control phase (typically labelled Phase A) and an intervention phase (typically labelled Phase B). By evaluating how an outcome measure changes for each phase, an indication of the ability for the intervention to cause an effect on the outcome measure is found [23].

**Figure 5:**
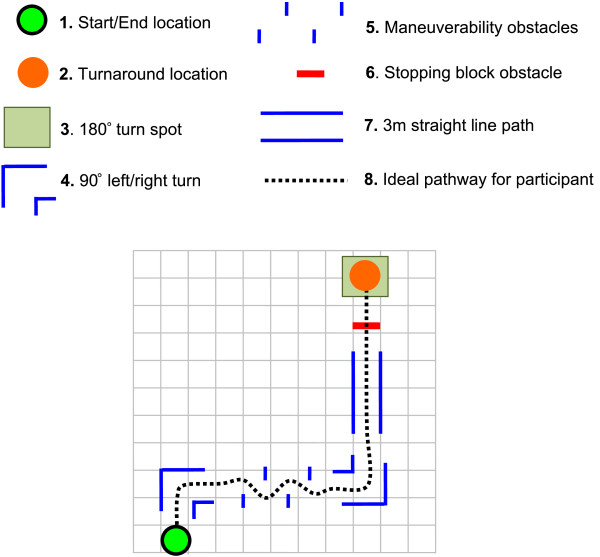
**Sample obstacle course for user trials.** Each grid square represents a 1 m × 1 m zone. All obstacles, except the 180° turn (no walls), were built using 5 cm thick and 1.2 m high foam walls. For the stopping block the participant had to stop within 0.5 m of the obstacle before it was removed from their path. The ideal path for the participant is marked by the dotted line. Participants performed a 180° turn at the turnaround location to re-enter the course and manoeuvre back to the start.

For this evaluation, participants were asked to drive a powered wheelchair (Pride Mobility™ Quantum 6000z) through an obstacle course (Figure 5) under two phases: Phase A) driving without the IWS, and Phase B) driving with the IWS. The ordering of the phases was randomized in order to negate any learning or fatigue effects. In each phase the participants completed the course (or runs) five times, with one run occurring once a day (that is, the total phase lasted five days). The obstacle course was composed of six essential movements related to powered wheelchair use. These movements were derived from the Wheelchair Skills Test (WST) [24] and the Power-Indoor Mobility Driving Assessment (PIDA) [25]. Both are clinical assessments for powered wheelchair mobility. The movements in the obstacle course included: 1) 90° left turn, 2) 90° right turn, 3) 3 m straight path, 4) weaving around obstacles, 5) 180° turn, and 6) stopping. All movements were driven through twice per run, with the exception of the 180° turn, which was driven through once per run (Figure 5). To further minimize the effects of testing bias, the order of the movements within the course was randomized for each run in the study.

Participants were recruited from a long-term care institution according to the following inclusion criteria: above age 60; consent from their substitute decision maker; minimal experience with a powered wheelchair (to minimize historic effects); mild-to-moderate cognitive impairment (typically scored as 11-26/30 on the Mini-Mental State Exam, a screening test for cognitive impairment) [26]; able to identify joystick directions; able to speak English; and no history of aggression. Five participants were recruited and enrolled following ethics approval from the University of Toronto Research Ethics Board. Two participants completed all 10 runs within the study. The other three participants withdrew from the study due to various reasons: scheduling conflicts, disinterest in the study, and an unrelated health problem. Participant 1 was age 69, had 6 hours of previous driving experience, and had a MMSE score of 25 (mild cognitive impairment). Participant 2 was age 62, had no previous driving experience, and had a MMSE score of 13 (moderate cognitive impairment).

Training occurred before each phase for each participant until they demonstrated cause-effect understanding between joystick directions and wheelchair movements. This training was reiterated before each run if the participant had trouble demonstrating this understanding in a short re-evaluation period before the run (i.e., asking the participants to drive the wheelchair left/right/forward/backwards and seeing their response). For Phase B, a demonstration of how the IWS operated was performed before each run.

#### ***Outcome measures***

Outcome measures were divided into two categories: safety and usability. Both categories had objective and subjective components of the measures (Table 2). Objective safety measures comprised of the ability to complete an essential movement without a collision (named “movement pass rate”) and the number of collisions that occurred within the sensor’s field of view (FOV collisions). Objective usability measures included: the time taken to complete the obstacle course run, and the participant’s adherence to audio prompts (that is, if they moved the joystick into the prompted direction within the first three joystick motions after the prompt). The three motion limit was used to allow for slight user compensation if they encountered any joystick dead-zone.

**Table 2:** Summary of the outcome measures used for user trials

	**Measurement category**	
	**Safety**	**Usability**
**Objective component**	-Movement pass rate (ability to complete essential movements without a collision).	-Time to complete obstacle course run.
	-Number of collisions (FOV).	-Adherence to audio prompts.
**Subjective component**		-NASA-TLX (Task Load Index) score.
	-QUEST 2.0: safety item.	-QUEST 2.0: simplicity of use item.

Note: QUEST 2.0 total score was also used to gauge overall user satisfaction of the device.

Two subjective tests were administered to the participants: the Quebec User Evaluation of Satisfaction with assistive Technology (QUEST 2.0) [27] and the NASA Task Load Index (NASA-TLX ) [28].

QUEST 2.0 is an outcome measure related to user satisfaction of assistive devices and it is comprised of 12 satisfaction items. However, only eight were relevant for this study (the other scores relate to servicing of the device). The relevant satisfaction items were the device’s: 1) dimension, 2) weight, 3) adjustments, 4) safety, 5) durability, 6) simplicity of use, 7) comfort, and 8) effectiveness. Each item was graded by the user through a 5-point Likert item ranging from “not satisfied at all” (1.0) to “very satisfied” (5.0). A total device score indicates overall device satisfaction and was calculated by averaging the eight categories. To examine subjective safety and usability, the specific QUEST 2.0 item ratings for safety and simplicity of use were noted.

Psychometric properties of QUEST 2.0 are reported in the QUEST manual [29]. Test-retest stability was found to be at moderate to substantial levels of agreement (calculated by weighted kappas). Inter-rater reproducibility ranged from fair to substantial agreement (calculated by weighted kappas). Internal consistency was found to be very good (calculated by Cronbach alpha coefficient). Content validity of QUEST was evaluated by 12 international experts, and construct validity was determined by factor analysis of data collected from 150 mobility device users.

The NASA-TLX is a measure of workload imposed by a given task and relates to subjective usability. It is comprised of six dimensions related to the workload demands on the user and the user’s interaction with the task. These dimensions are: 1) mental demand (perceived mental activity required); 2) physical demand (perceived physical activity required); 3) temporal demand (perceived time pressure related to the task); 4) performance (how successful the user felt in accomplishing the goals of the task); 5) effort (how hard the user felt they had to work to achieve their performance); and 6) frustration (how insecure, irritated, discouraged, or stressed the user felt when performing the task). For this study, the task was defined as: manoeuvring a powered wheelchair through an obstacle course with as few collisions as possible. Participants rated each dimension from 0 (minimal workload/good performance) to 20 (high workload/bad performance). A simplified total workload score can be calculated by summing the dimensions together [30]. The NASA-TLX is sensitive to different tasks [28]. It also has a very high convergent validity (Pearson Correlation Coefficients > 0.97, p < 0.001) and the best concurrent validity when compared to other measures of global mental workload (Subjective Workload Assessment Technique – SWAT, and Workload Profile – WP) [31]. As well, it has been used to assess older adults under various driving tasks [32,33].

All measures were taken during each run, with the exception of the QUEST 2.0, which was administered after each phase. As is typical with single-subject research, the results were analyzed for each participant separately. Before conducting visual analysis, data points from all runs were checked to ensure no serial dependency using Bartlett’s test since serially dependent data are known to cause errors in visual analysis [23]. Movement pass rates were summed over each phase and compared between the phases. Adherence to audio prompts was calculated as a percentage of all correct audio prompts given to the participant in the IWS phase. Collision and time data were plotted and analyzed descriptively. Results from the QUEST 2.0 and NASA-TLX (averaged per phase) were compared between each phase.

## Results

### Stage one: environment of use

**Figure 6:**
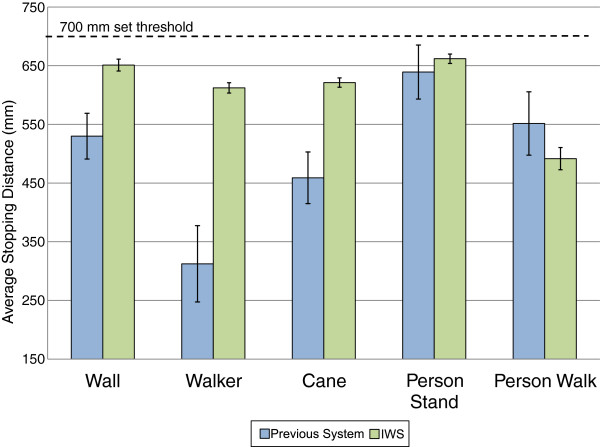
**Average stopping distances from the obstacle to the sensor.** Dashed line indicates set threshold distance (700 mm). Results for the previous iteration of the IWS are shown in blue^1^, and the current IWS are shown in green. Distances that are closer to the threshold indicate a more accurate stopping distance. Error bars show the standard deviation of stopping distances.

The IWS performed successfully in all anti-collision test conditions. Table 3 compares the anti-collision performance of our previous system [21] and the IWS. Two misses occurred in both the wall and cane scenarios with the previous system. Figure 6 summarizes the average stopping from the object to the sensor^a^. In all but one condition, the IWS had a closer stopping average to the set threshold than the previous system. Only the moving person scenario was on average 60 mm farther from the threshold than the previous system. For all conditions the IWS had smaller standard deviations in stopping distances (ranging from 16 - 37 mm) than the previous system (ranging from 78 -108 mm).

**Table 3:** Comparison of the previous IATSL system and the IWS anti-collision performance

**Test condition**	**Hits**		**Misses**		**False alarms**		**Correct rejects**	
	**Prev.**	**IWS**	**Prev.**	**IWS**	**Prev.**	**IWS**	**Prev.**	**IWS**
No object	0	0	0	0	0	0	20	20
Wall	18	20	2	0	0	0	0	0
Walker	20	20	0	0	0	0	0	0
Cane	18	20	2	0	0	0	0	0
Person stand	20	20	0	0	0	0	0	0
Person walk	20	20	0	0	0	0	0	0
Total	96	100	4	0	0	0	20	20

Table 4 describes the navigation performance of the previous system and the IWS. Both had successful prompting results in all navigation scenarios.

**Table 4:** Comparison of the previous IATSL system and the IWS navigation performance

**Test condition**	**Hits**		**Misses**		**False alarms**		**Correct rejects**	
	**Prev.**	**IWS**	**Prev.**	**IWS**	**Prev.**	**IWS**	**Prev.**	**IWS**
No object	0	0	0	0	0	0	20	20
Object left	20	20	0	0	0	0	0	0
Object right	20	20	0	0	0	0	0	0
Total	40	40	0	0	0	0	20	20

### Stage two: user trials

**Figure 7:**
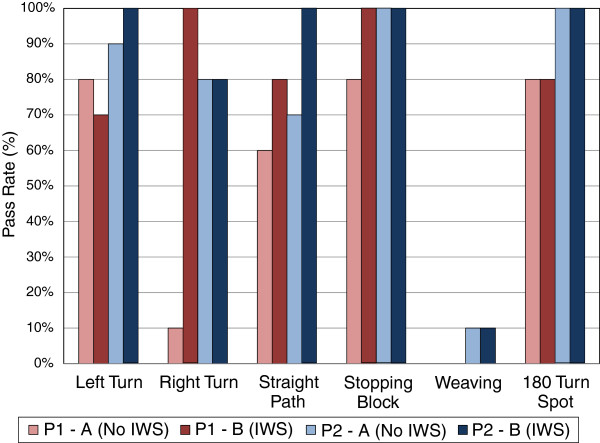
**Movement pass rate for participants in each phase.** N = 10 for all movements except the 180° turn spot where N = 5.

**Figure 8:**
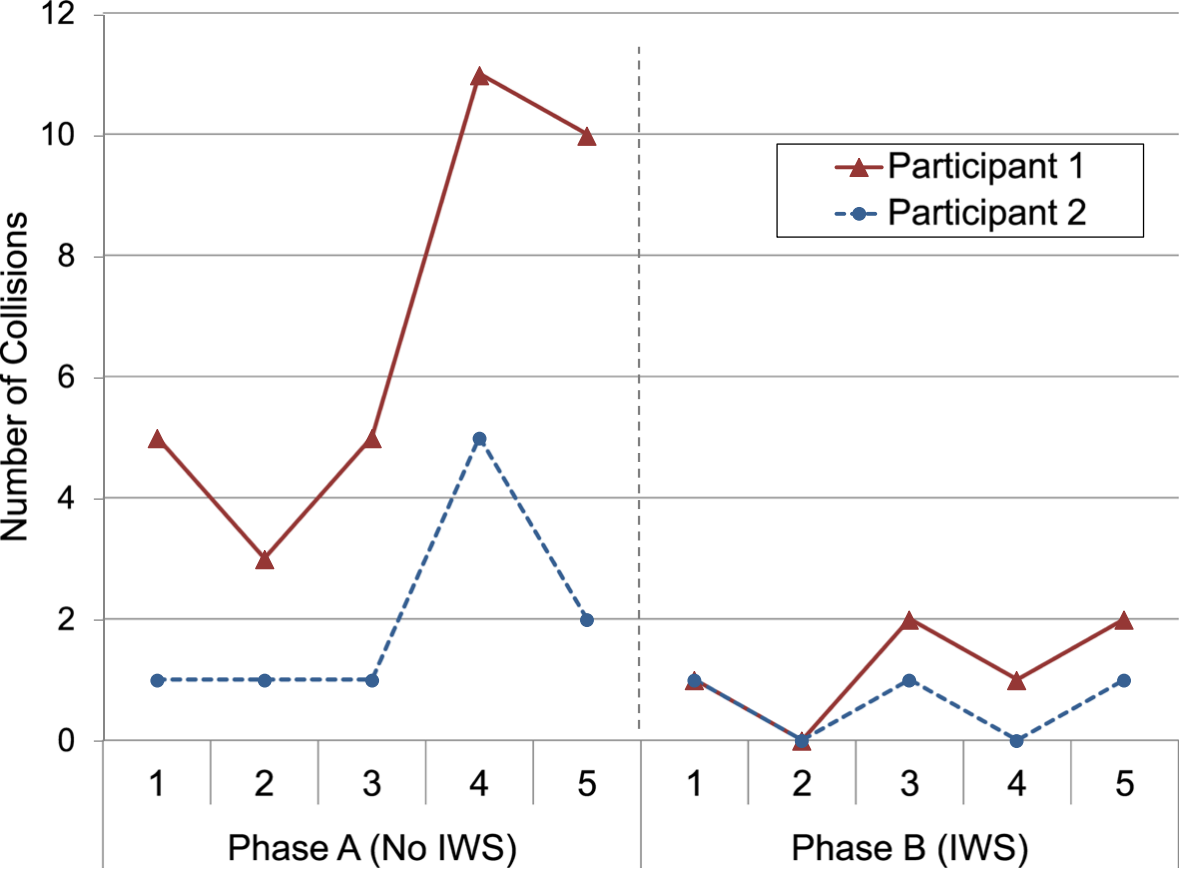
FOV (field of view) collision for participants in each run.

**Figure 9:**
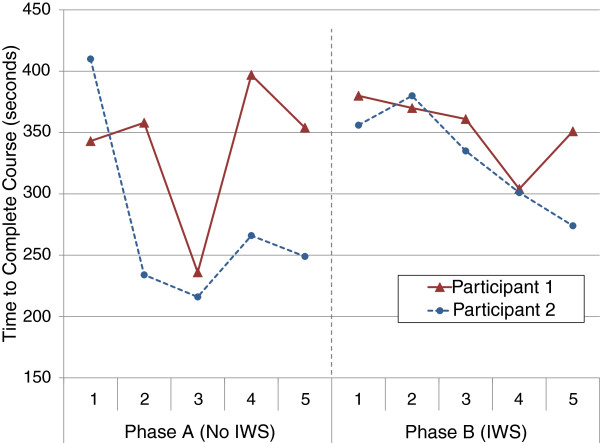
Time taken to complete the obstacle course for participants in each run.

For objective measures: Figure 7, shows the movement pass rates totalled across each of the participant’s phases. A change in pass rate occurred between the phases for participant 1’s left turn (80% to 70%), right turn (10% to 100%), straight path (60% to 80%), and stopping (80% to 100%); and participant 2’s left turn (90% to 100%), and straight path (70% to 100%). The number of FOV collisions and the time to complete each run are shown in Figures 8 and 9 respectively for both participants. Participant 1’s FOV collisions had a sharp discontinuity when the IWS was introduced (one of the criteria to support the impact of an intervention in single-subject research [23]), and both participants maintained a lower magnitude of collisions during the IWS phase. It is noted that a peak in collisions occurred in both participant 1 and 2’s run 4. For participant 1’s time to completion, there was no sharp discontinuity between the phases, and the average in each phase is similar; whereas participant 2’s time to completion shows a rise in time when the IWS was introduced, and also a trend of decreasing time in each phase. Both participants did not reach a high adherence to audio prompts: participant 1 had an adherence of 76.5% and participant 2 had an adherence of 56.4%.

**Figure 10:**
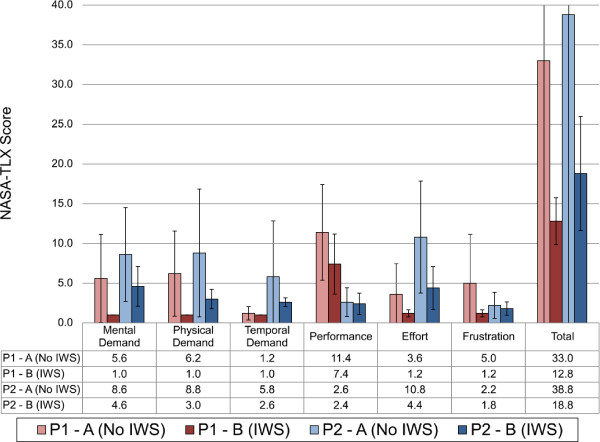
**Average NASA-TLX scores for participants in each phase.** Standard deviation is shown in error bars for each score.

For subjective measures: Table 5 and Figure 10 report the scores for QUEST 2.0 and the NASA-TLX (averaged across each phase) respectively. In most cases the QUEST 2.0 scores for satisfaction increased with the IWS intervention. The exception to this was participant 2’s simplicity of use and total device score, which remained the same between the two phases. In all cases, the average NASA-TLX scores were lower with the IWS.

**Table 5:** Summary QUEST 2.0 satisfaction scores for participants in each phase

**Satisfaction item**	**P1-A**	**P1-B**	**P2-A**	**P2-B**
	**(No IWS)**	**(IWS)**	**(No IWS)**	**(IWS)**
Safety	2.0	4.0	4.0	5.0
Simplicity of use	1.0	3.0	5.0	5.0
Total device	2.5	3.5	4.9	4.9

Scores range from not satisfied at all (1.0), to very satisfied (5.0). The Total Device score (average of the 8 QUEST 2.0 device components) and specific components of Safety and Simplicity of Use are listed (see “Methods, Stage Two: User Trials, Outcome Measures”).

## Discussion

### Stage one: environment of use

The IWS has the potential to perform well in an institutional setting as it enabled completion of all anti-collision and navigation scenarios successfully. When compared to our previous iteration [21], the IWS has improved detection of obstacles (for example, wall, and cane) and improved accuracy in stopping distances (that is, lower standard deviation in stopping distances). The improvement in detection of obstacles can be attributed to the higher sensor resolution – which is able to detect smaller objects; and the new blob algorithm – which is able to bound small profile objects with high sensitivity due to its noise rejection methods. Improvement in stopping distance accuracy is attributed to the higher update rate of the new system. Stopping distance accuracy is important, as it means the system will be more consistent to respond to objects in its environment. Having a high variability in stopping distance corresponds to unpredictability in anti-collision performance during real-world use. Although both the previous system and the IWS performed the same in navigation scenarios, it is expected that the IWS will be able to handle complex scenes with greater accuracy due to its higher resolution occupancy grid. In terms of the deviation of stopping distances from the set threshold, only the moving person scenario performed worse with the IWS. An explanation for this is that when a large object (for example, a person) abruptly enters the FOV of the sensor, the algorithm to bound regions of high disparity is computationally slowed down because of the size of the object. When compared to the standing person scenario, the IWS outperformed the previous system because the closest point of the person (for example, the hand) can be identified and bounded quickly. Overall, the improvements made to the system show promising results. Future work can focus on increasing the system’s speed and accuracy for correctly responding to obstacles in the environment.

### Limitations of stage one

Although the IWS performed well, the evaluation was conducted in a controlled environment without natural light. It is expected that natural light will affect the accuracy of obstacle detection. The developed blob detection algorithm is able withstand small lighting noise; however it is still susceptible to whiteout, which is an inherent difficulty for vision sensors.

Another issue is that this evaluation covered a limited number of scenarios. Other common activities such as table docking, bedside transfer, and doorway entry should be designed for and evaluated in future iterations.

Lastly, trials were conducted at a slow velocity (0.16 m/s) to allow for comparison to the previous system [21]. It is expected that the improvement in the update rate of the new system allows for higher velocities to be supported for collision detection. In future work, the effect of increasing wheelchair velocity on anti-collision performance should be explored.

### Stage two: user trials

Participant 1’s results support the idea that safety of powered wheelchair use can be improved with the IWS. Results from the movement pass rates show a marked increase in the right turn task (10% to 100%), which demonstrates that the IWS has the potential to help the user complete essential movements safely. Although there were changes in other pass rates between phases, these differences are not conclusive and may also be attributed to day-to-day driving variability. There was also a noticeable reduction in FOV collisions when the IWS was introduced, again supporting the idea of increased safety. As well, from a subjective viewpoint participant 1 felt more satisfied with safety when the IWS was used. Although these are positive remarks, it is important to note that not all FOV collisions were prevented by the IWS and the participant’s pass rates with the IWS were not 100% in all cases (also due to the IWS’s inability to prevent side/rear collisions). Finally, the sharp increase in collisions for participant 1’s phase A, run 4, could be attributed to a bad driving day, which in turn could be caused by other factors such as tiredness.

In terms of the IWS’s usability for participant 1, from an objective standpoint there was little improvement when the IWS was introduced because the time to complete the course did not show a change between phases. For subjective usability, QUEST 2.0 and NASA-TLX results showed an improvement, which is an interesting contrast to the objective result. The QUEST 2.0 simplicity of use score increased from “not satisfied at all” (1.0) to “more or less satisfied” (3.0), which is a positive note, yet also suggests that usability can be further improved for this participant. NASA-TLX results suggest that the IWS helped lower the perceived workload for this participant; however it is noted that this perceived improvement did not translate into improved objective performance with the powered wheelchair. Objective performance was inferred to be supported by the navigation feature of the IWS (that is, participants would remain stopped at obstacles for less time due to navigation prompts).

Participant 2 also showed an improvement in safety when the IWS was used. For movement pass rates, a significant increase in the straight path was seen (70% to 100%). Although FOV collisions were not observed to have sharp change between phases, it is inferred that the IWS can limit the severity of bad driving days (for example, participant 2’s phase A, run 4) by limiting the number of collisions that occur. Satisfaction with safety (QUEST 2.0) also improved when the IWS was used, but not all collisions were prevented with the IWS.

For usability, participant 2’s objective usability was negatively affected with the intervention of the IWS; however the participant had a trend of improvement in later runs, which suggests a learning effect for using the system better. Subjectively, QUEST 2.0 scores remained high with the IWS, and NASA-TLX results suggest that the IWS can lower the perceived workload for this participant. It is noted that the subjective usability was improved even though objective usability decreased. In an ideal case, both objective and subjective usability would be improved by the IWS. The powered wheelchair should be perceived easy to use by the user and also efficient to manoeuvre around the environment. The decrease in objective usability is likely linked to prompting adherence, as participant 2 had a low adherence (56.4%) for following the prompts.

### Limitations of stage two

Due to the limitations of single-subject research designs, the small sample size, and the selection criteria used in this study – it is difficult to generalize these results to the entire population of cognitively impaired older adults. These results nevertheless give insight into how the IWS can affect members within the desired user population and how our system’s design might be improved.

The trials, with three participants not able to complete the full study, also highlight the difficulties with conducting multi-day evaluation studies with this population. Future researchers should consider the time necessary for evaluation studies, or identify alternative study designs to involve users, as there are unique challenges with the cognitively impaired older adult population and the institutional setting [34,35].

Because of participant drop out, the effects of learning to drive a powered wheelchair could not be negated through the phases as originally designed. Participants with a B-A phase ordering did not complete this study. In future research, a study with an A-B-A design could help mitigate the learning effect issue. Despite this limitation, there were other visual indicators of the intervention effect which have been described in the discussion section.

It is also acknowledged that the validity of subjective ratings given by individuals with cognitive impairments may be unclear. In this study, it was deemed that the participants demonstrated sufficient understanding of the subjective safety questions as their answers were corroborated by the objective results (for example, participants scores on subjective safety improved as objective performance of safety improved). Subjective usability results can be interpreted with caution. For future studies, subjective response validity could be improved by using observational data and open-ended questions during trials as additional corroborative evidence. Even though there is difficulty with validating subjective ratings with this population, subjective responses should still be investigated as it is an important aspect of assistive technology use.

### Remarks on safety and usability

The IWS has the potential to improve safety of powered wheelchair driving by lowering the number of collisions that can occur and by helping individuals to complete essential movements safely. Because the participants drove through a structured obstacle course based on movements related to daily life, it is also easier to extrapolate how the IWS would affect daily powered wheelchair driving. At this time, the IWS will likely perform well to help users navigate through hallways and around large rooms, but will have difficulty weaving through crowds of people or objects successfully. Previous groups have evaluated their intelligent/smart wheelchair with obstacle courses, but some of these courses were small and only cover a few movements related to powered wheelchair driving in daily life [12,14]. This study highlights the need for the movements included in obstacle courses to be justified, relevant, and as encompassing of movements encountered in daily life as possible. Due to limitations of the IWS (only frontal sensor detection), not all movements of the Wheelchair Skills Test and Powered-Indoor Driving Assessment were included in the course as the system would have no impact on some of these movements. However, the Wheelchair Skills Test has been proposed as a standard evaluation course for intelligent wheelchairs by a group from McGill University [36]. Proper movement evaluation of intelligent wheelchairs will give insight into areas of improvement that might otherwise be missed, and a standard measure would allow for objective comparison between wheelchair designs. In this study, the weaving motion from the Powered-Indoor Driving Assessment identified the need for improvement of the IWS’s anti-collision feature when tightly turning around obstacles.

Although the Wheelchair Skills Test presents a methodology to evaluate common movements with intelligent wheelchairs, there is still no standard method of comparing intelligent wheelchairs and their response to dynamic obstacles. The structure of our environmental trials could be a precursor to such an evaluation, as it tested both static and dynamic obstacles. Yet, it should be noted that additional object scenarios in the environmental trials will be beneficial for comprehensive testing.

Upon further analysis of the collisions that occurred with the IWS (that is, the failures in movement pass rate), it was found that there were two main issues. The first, was that the IWS lacked sensor coverage in the side and rear areas of the powered wheelchair, which allowed collisions to occur in these regions. The second was that the stereovision camera had a blind spot at close proximity (~500 mm of sensor). Combined with the lack of full sensor coverage around the wheelchair, obstacles could enter into this blind spot and cause a collision when the wheelchair turned into them at close proximity. Future work will involve eliminating these collisions by extending sensor coverage around the wheelchair.

Usability of the powered wheelchair was an interesting issue. Although both participants showed improvements subjectively with the IWS, the objective performance of powered wheelchair use did not increase substantially. One possible explanation for the subjective improvements is that the IWS’s ability to prevent collisions and offer navigation tips gave the users assurance that lowered their perceived driving demands. However, the lack of objective improvement shows that navigation assistance of the IWS did not produce its desired effect. When linked with the low joystick adherence observed, it is clear that the IWS can still be improved for usability. The difference in participant prompting adherences suggests that audio prompting may be more effective for certain individuals. Perhaps prompting methods that are tailored to individual preferences (e.g., audio, visual, haptic feedback [37]) would be useful in increasing adherence and user proficiency of navigating the environment. This is an important point because users who navigate poorly in their environment can be seen as a hindrance to others [38].

System customizability could also extend beyond inter-person variability (differences in preferences and ability), to intra-person day-to-day variability. In the user trials, both participants had a bad driving day compared to other trials of the same phase. The occurrence of such a day could be due to a number of factors, such as changing alertness throughout the day or emotional/cognitive distraction. Regardless of the reason, the existence of day-to-day variability should be accounted for. One way to account for both inter-person and intra-person variability is to have multiple levels of assistance given by the IWS. In this way, the wheelchair could be set to a level of assistance that matches the user’s retained abilities.

## Conclusions

This research describes the Intelligent Wheelchair System (IWS) that was developed to help older adults with cognitive impairments drive a powered wheelchair safely and effectively. An evaluation of the system was conducted in two stages and examined the IWS with an environment of use and with members of its desired user population. The IWS’s anti-collision and navigation features performed successfully during simulated environment trials, and user trials showed the IWS’s potential to improve power wheelchair safety and subjective usability. Overall, the structure and detail of the trials helped expose system issues that were not apparent during the conceptualization of the system (for example, difficulty with weaving movements, low prompting adherence, and prompting customization to individuals). This highlights the importance of user evaluation in the design of intelligent wheelchairs. Lessons from this project suggest that an intelligent wheelchair for older adults with cognitive impairments should be customizable to the changing needs of the individual. Multiple levels of assistance that can be adapted to the user’s level of ability and day-to-day variability will be beneficial for an aging population.

## Endnotes

^a^Distances for the previous system have been offset by 45 mm to take into account sensor mounting. The previously reported results measured the distance between the object to the front of the wheelchair [21].

## Competing interests

The authors declare that they have no competing interests.

## Authors’ contributions

TVH developed the hardware/software of the IWS, formulated the methodologies for the clinical trials, performed data collection and analysis, and drafted the manuscript. RHW contributed to the methodologies and data collection of the clinical trials, and revised the manuscript. AM developed the methodologies for the environmental trials and revised the manuscript. All authors read and approved the final manuscript.
